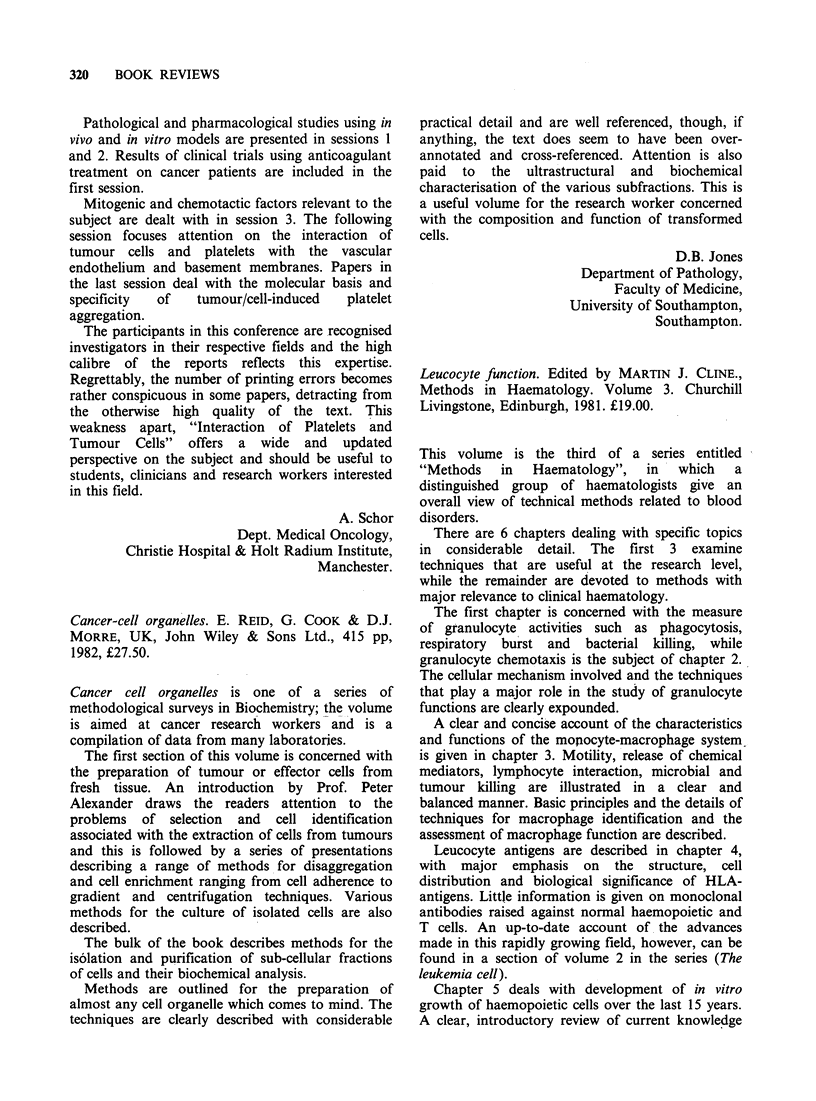# Cancer-cell organelles

**Published:** 1983-02

**Authors:** D.B. Jones


*Cancer-cell organelles*. E. Reid, G. Cook & D.J. Morre, UK, John Wiley & Sons Ltd., 415 pp, 1982, £27.50.

*Cancer cell organelles* is one of a series of methodological surveys in Biochemistry; the volume is aimed at cancer research workers and is a compilation of data from many laboratories.

The first section of this volume is concerned with the preparation of tumour or effector cells from fresh tissue. An introduction by Prof. Peter Alexander draws the readers attention to the problems of selection and cell identification associated with the extraction of cells from tumours and this is followed by a series of presentations describing a range of methods for disaggregation and cell enrichment ranging from cell adherence to gradient and centrifugation techniques. Various methods for the culture of isolated cells are also described.

The bulk of the book describes methods for the isolation and purification of sub-cellular fractions of cells and their biochemical analysis.

Methods are outlined for the preparation of almost any cell organelle which comes to mind. The techniques are clearly described with considerable practical detail and are well referenced, though, if anything, the text does seem to have been over-annotated and cross-referenced. Attention is also paid to the ultrastructural and biochemical characterisation of the various subfractions. This is a useful volume for the research worker concerned with the composition and function of transformed cells.

D.B. Jones
Department of Pathology,
Faculty of Medicine,
University of Southampton,
Southampton.